# Effect of Dietary Vanaspati Alone and in Combination with Stressors on Sero-biochemical Profile and Immunity in White Leghorn Layers

**DOI:** 10.4103/0971-6580.75850

**Published:** 2011

**Authors:** M. Alpha Raj, A. Gopala Reddy, A. Rajasekhar Reddy, K. Adilaxmamma

**Affiliations:** Department of Pharmacology and Toxicology, College of Veterinary Science, Rajendranagar, Hyderabad - 500 030, India; 1 Department of Pharmacology and Toxicology, College of Veterinary Science, Korutla - 505 326, India; 2 Department of Pharmacology and Toxicology, College of Veterinary Science, Tirupati - 517 502, India

**Keywords:** Immunity, layers, white leghorn, vanaspati

## Abstract

A total of 160 White Leghorns of 20 wk age were divided randomly into eight groups. Groups 1, 3, 4 and 5 were fed basal feed and the rest were fed 5% vanaspati supplemented feed until 42 wk of age. From 42 to 54 wk, groups 3, 4 and 5 were fed 1% ferrous sulfate, 100 ppm chlorpyrifos (CPS) and 100 ppm cadmium, respectively, along with basal feed and groups 6, 7 and 8 were fed similar stressors, respectively, along with 5% vanaspati. Groups 1 and 2 served as controls for basal feed and 5% vanaspati feed. Alkaline phosphatase (ALP), alanine transaminase (ALT), total protein, albumin, globulin, A/G ratio, total cholesterol, high density cholesterol (HDL), triglycerides, creatinine, hemagglutination inhibition (HI) titer, and phytohemagglutination (PHA) index were studied. Supplementation of vanaspati resulted in a significant reduction in PHA, cholesterol, albumin and HI titer. Cadmium significantly increased ALP, AST, creatinine and paradoxically increased HDL cholesterol and HI titers. Vanaspati along with cadmium showed similar effects. Administration of CPS lowered PHA index, whereas supplementation along with vanaspati decreased the HI titers and increased the PHA index. Supplementation of vanaspati alone and in combination revealed harmful effects and aggravated the toxicities of CPS and cadmium. Hence, it is concluded that consumption of vanaspati could be harmful.

## INTRODUCTION

Now-a-days, hydrogenated vegetable oils, which are solids at room temperature and have better physical properties, shelf life and flavor stability, are preferred over animal fats for mixing in feeds. However, the process of hydrogenation leads to the formation of *trans* fatty acids which cause many deleterious effects on health. In parts of India, *trans* fats from hydrogenated vegetable oil in the form of vanaspati are consumed in greater quantity than in the United States.[1,2] In India, cardiovascular diseases cause 3 million deaths per year, accounting for 25% of all mortality.[3] The World Health Organization predicted that deaths due to circulatory system diseases are projected to double between 1985 and 2015.[4] Dietary factors that may contribute to a high ischemic heart disease (IHD) risk in India include low intakes of vitamin B6 and folate[5] and high intakes of *trans* fatty acids, which have been associated with risk in studies conducted in the West.[6]

Further, a wide range of organic and inorganic compounds may occur in feedstuffs, including pesticides, industrial pollutants, radionuclides and heavy metals. Pesticides that may contaminate feeds originate from most of the major groups, including organochlorine, organophosphate and pyrethroid compounds.[7] The interaction of the contaminants with added fats was not addressed earlier.

In view of the increasing use of hydrogenated vegetable oil in both poultry and human diets, the present study was taken up to assess the effects of supplementation of vanaspati in layer diet alone and in combination with various stressors on sero-biochemical and immunological parameters.

## MATERIALS AND METHODS

A total of 160 White Leghorn pullets, aged 18 wk, were procured from a local farm and were housed in the poultry Layer House of College of Veterinary Science, Rajendranagar, Hyderabad, with 16 hours of light. The birds were given standard layer feed formulated by the Department of Poultry Science, College of Veterinary Science, Rajendranagar and *ad libitum* water.

### Feed composition

The feed was formulated using the following ingredients (for 100 kg): maize 52 kg; soyabean 20 kg; sunflowercake 12 kg; de-oiled rice bran 6 kg; shell grit 8 kg; di-calcium phosphate 1.33 kg; trace minerals 100 g; vitamins A, B1, D3 – 15 g; vitamin B12 – 250 g; salt 333 g; digestible crude protein (DCP) 18.70%; metabolizable energy (ME) 2648 kcal/kg; calcium 3.67%; available phosphorus 0.69%; lysine 0.91% and methionine 0.32%. The feed supplemented with 5% vanaspati had an energy content of 2965 kcal/kg.

### Chemicals

Vanaspati (Dalda; Bunge Pvt. Ltd., Mumbai, India), chlorpyrifos EC 20% (Dursban; NOCIL, Pvt. Ltd., Mumbai), ferrous sulfate hepta hydrate and cadmium chloride (Qualigens Fine Chemicals, Mumbai), New castle disease virus (NDV) vaccine, R2B strain from Indovax, Indovet Pvt. Ltd. New Delhi. All the chemicals used were procured from Qualigens Pvt. Ltd. and were of analytical grade.

### Experimental design

After an acclimatization period of 2 wk, the experiment was started with the birds attaining an age of 20 wk and the birds were divided into eight groups of 20 birds each as follows.

Group 1: Basal feed (20–54 wk)

Group 2: Vanaspati feed (5%) (20–54 wk)

Group 3: Basal feed (20–54 wk) + 1% ferrous sulfate (42–54 wk)

Group 4: Basal feed (20–54 wk) + 100 ppm chlorpyrifos (42–54 wk)

Group 5: Basal feed (20-54 wk) + 100 ppm cadmium (42-54 wk)

Group 6: Vanaspati feed (20–54 wk) + 1% ferrous sulfate(42–54 wk)

Group 7: Vanaspati feed (20–54 wk) + 100 ppm chlorpyrifos (42–54 wk)

Group 8: Vanaspati feed (20–54 wk) + 100 ppm cadmium (42–54 wk)

Birds of all the groups were vaccinated with NDV vaccine R2B at 46 wk of age and a booster subsequently after 21 days. The period from 38 to 42 wk, from 42 to 46 wk, from 46 to 50 wk and from 50 to 54 wk was considered as months 0, 1, 2 and 3, respectively.

Blood samples were collected from the jugular vein at the end of every month from 10 birds in each group and serum was separated for the estimation of alkaline phosphatase (ALP), alanine transaminase (ALT), total protein, albumin, globulin, A/G ratio, total cholesterol, high density lipoprotein (HDL), triglycerides and creatinine, using standard kits supplied by Qualigens Pvt. Ltd. Parameters like globulins and A/G ratio were calculated by using the values from relevant parameters. HI titer[8] and PHA index[9] were estimated at the end of the experimental period.

### Statistical analysis

The data for sero-biochemical parameters were analyzed by two-way analysis of variance (ANOVA) followed by Tukey’s *post hoc* test, and the data for immunological parameters were analyzed by one-way ANOVA using Statistical Package for Social Sciences (SPSS) 15^th^ version. The significance value was set at *P*<0.05. Logarithmic transformation was used to the correct non-normal data and Welch ANOVA was used if the assumption of homogeneity of variance was found to be violated.

## RESULTS AND DISCUSSION

The results of sero-biochemical profile and immunological parameters are depicted in Tables 1–3, respectively.

**Table 1:** Marginal means of sero-biochemical parameters in different groups

Group	Cholesterol (mg/dl)	HDL (mg/dl)	Triglycerides (mg/dl)	Alkaline phosphatase (IU/l)	ALT (IU/l)	Creatinine (mg/dl)
A								
Normal	111.19±1.06^b^	200.36±46.80^a^	630.79±1.14^b^	200.36±46.80^a^	10.76±0.84^a^	0.40±0.07^ab^
Vanaspati	103.19±1.06^ab^	315.17±46.18^abc^	669.24±1.13^b^	315.17±46.18^abc^	14.67±0.84^bc^	0.36±0.07^a^
Ferrous sulfate	99.23±1.06^ab^	267.97±47.50^ab^	455.65±1.13^b^	267.97±47.50^ab^	15.08±0.84^bc^	0.48±0.07^ae^
Chlorpyrifos	108.88±1.07^ab^	445.41±46.18^bc^	600.74±1.14^b^	445.41±46.18^bc^	11.95±0.84^ab^	0.40±0.07^ad^
Cadmium	104.01±1.06^ab^	672.76±46.18^d^	243.48±1.13^a^	672.76±46.18^d^	18.50±0.84^c^	0.58±0.07^de^
Ferrous sulfate + vanaspati	89.81±1.06^ab^	524.89±49.30^c^	406.72±1.14^ab^	524.89±49.30^c^	13.51±0.84^bc^	0.48±0.07^bcde^
Chlorpyrifos + vanaspati	91.38±1.06^ab^	262.48±47.50^ab^	660.60±1.13^b^	262.48±47.50^ab^	14.99±0.84^bc^	0.41±0.07^ac^
Cadmium + vanaspati	84.43±1.06^a^	613.40±46.18^d^	242.78±1.13^a^	613.40±46.18^d^	18.22±0.84^c^	0.64±0.07^de^
B								
Vanaspati	99.22±3.64	10.69±1.26^NS^	600.05±31.57^NS^	323.65±25.55^NS^	14.15±2.74^NS^	0.47±0.04^NS^
Normal	114.96±3.69*	11.57±1.28^NS^	611.77±32.00^NS^	296.68±25.27^NS^	20.62±2.80^NS^	0.46±0.04^NS^

A: effect of individual treatment; B: overall effect of vanaspati supplementation; Values are Mean±SE. Two-way ANOVA followed by Tukey’s Honestly Significant Difference (HSD) *post hoc* test; Means with different superscripts are significantly different (*P*<0.05); ALT, alanine transaminase;

* Significantly different (*P*<0.05)

**Table 2:** Marginal means of sero-biochemical parameters in different groups

Group	Protein (g/dl)	Albumin (g/dl)	Globulin (g/dl)	A/G ratio
A				
Normal	6.05±1.04^NS^	1.70±0.04^NS^	4.34±1.05^NS^	0.40±0.02^NS^
Vanaspai	6.01±1.04^NS^	1.65±0.04^NS^	4.33±1.05^NS^	0.40±0.02^NS^
Ferrous sulfate	5.37±1.04^NS^	1.63±0.04^NS^	3.70±1.05^NS^	0.46±0.02^NS^
Chlorpyrifos	5.89±1.04^NS^	1.68±0.04^NS^	4.20±1.06^NS^	0.40±0.02^NS^
Cadmium	5.95±1.04^NS^	1.73±0.04^NS^	4.20±1.05^NS^	0.42±0.02^NS^
Ferrous sulfate + vanaspai	5.51±1.04^NS^	1.58±0.04^NS^	3.91±1.05^NS^	0.41±0.02^NS^
Chlorpyrifos + vanaspai	5.77±1.04^NS^	1.61±0.04^NS^	4.12±1.05^NS^	0.41±0.02^NS^
Cadmium + vanaspai	5.32±1.04^NS^	1.56±0.04^NS^	3.75±1.05^NS^	0.42±0.02^NS^
B				
Vanaspai	5.83±0.013^NS^	1.60±0.02	4.23±0.13^NS^	0.41±0.10^NS^
Normal	5.96±0.014^NS^	1.69±0.02^*^	4.27±0.13^NS^	0.42±0.10^NS^

A: effect of individual treatment; B: overall effect of vanaspati supplementation.; Values are Mean±SE. Two-way ANOVA followed by Tukey’s HSD *post hoc* test; Means with different superscripts are significantly different (*P*<0.05)

**Table 3:** Marginal means of immunological parameters in different groups

Group	HI (log_2_)	PHA index (mm)
A		
Normal	424.00±103.29^b^	0.43±0.05^ab^
Vanaspai	69.00±30.64^a^	0.65±0.06^bcd^
Ferrous sulfate	432.00±13.13^b^	0.47±0.09^ac^
Chlorpyrifos	512.00±125.69^b^	0.42±0.10^a^
Cadmium	1536.00±193.52^c^	0.63±0.09^bcd^
Ferrous sulfate + vanaspai	62.00±11.49^a^	0.75±0.18^bcd^
Chlorpyrifos + vanaspai	114.00±25.20^a^	0.82±0.06^d^
Cadmium + vanaspai	82.50±18.68^a^	0.56±0.09^ad^
B		
Vanaspai	81.86±11.35	0.69±0.06^*^
Normal	726.00±107.96^*^	0.49±0.04

A: effect of individual treatment; B: overall effect of vanaspati supplementation; Values are Mean±SE. Two-way ANOVA followed by Tukey’s HSD *post hoc* test; Means with different superscripts are significantly different (*P*<0.05); HI, hemagglutination inhibition; PHA, phytohemagglutination

In the present study, the activities of ALT and ALP were determined to assess the degree of damage to the liver as the levels of certain enzymes like ALT, AST, gamma glutamyl transferase (GGT), etc., are shown to be elevated following hepatocellular injury.[10] In this study, the activities of ALT and ALP were significantly elevated in the cadmium toxic control group, whereas ALT activity was elevated in ferrous sulfate group, suggesting the hepatocellular insult following administration of cadmium and ferrous sulfate. The supplementation of vanaspati with ferrous sulfate resulted in an elevated activity of ALP. The results are in accordance with those of Demerdesh *et al*.,[11] Kara *et al*,[12] and Yadav *et al*.,[13] who reported an increase in the activities of ALT and ALP in plasma following cadmium toxicity.

An increase in the total cholesterol, triglycerides and low density lipoprotein (LDL) with the decrease in HDL in serum will serve as a biomarker for hepatopathy, cardiac damage as well as renal failure.[10] In the present study, no changes in the serum cholesterol profile and protein profile were detected except that cadmium paradoxically caused a significant elevation of HDL and significant reduction in triglycerides. CPS produced a non-significant decrease in HDL and increase in total lipids of liver. Supplementation of vanaspati along with the stressors showed no effect on the above parameters except that HDL was increased in cadmium group. The overall effect of vanaspati supplementation revealed a significantly decreased serum cholesterol and albumin.

The increase in HDL and decrease in triglyceride concentration of cadmium group is attributed to the possible interference of cadmium with the test procedures. HDL was, however, low in CPS group, which is explained by the fact that HDL that is associated with paraoxonase enzyme, which helps in breaking down organophosphorus (OP) pesticides[14] and hence is bound to CPS oxon.[15] The supplementation of vanaspati resulted in decreased serum cholesterol level. Such a reduction in cholesterol level is attributed to the significant amounts of polyunsaturated fatty acids (PUFAs) present in vanaspati, despite hydrogenation, which gets incorporated into and consequently weakens the cell membranes when insufficient saturated fats are not available. This leads to deposition of cholesterol to strengthen the cell membranes and consequently results in lowering of blood cholesterol.[16] It was observed that the albumin level was reduced upon supplementation of vanaspati. This is due to fact that fatty acids are transported in a bound form with albumin in the blood stream.[17] In the present study, albumin was estimated by dye binding method and hence the method could not be used to estimate fatty acid bound albumin.

Non-protein nitrogenous (NPN) substances, such as serum creatinine, are increased only when renal function is below 30% of its original capacity in birds. In the present study, creatinine was significantly increased in the cadmium group and supplementation of vanaspati had no effect. The increased creatinine levels in serum indicate kidney damage. The elevated levels of creatinine are due to the damage caused by cadmium to the hepatic and renal tissue, leading to the liberation of marker enzymes into serum. The results are in accordance with those of Kara *et al*.,[12] and Yadav *et al*.[13]

In the present study, stressors showed little effect upon immunity. Paradoxically, cadmium showed a significant increase in antibody titer. A similar paradoxical increase in antibody titer was reported by Robohm.[18] However, it is assumed that the increase in antibody titer is probably a case of interference of cadmium with hemagglutination of RBC because the corresponding increase in globulin level of serum was not observed. The overall supplementation of vanaspati reduced HI titer. The PHA index, which is a measure of cell mediated immunity and T-cell proliferation, was significantly increased in vanaspati fed groups. This is probably due to the fact that higher intakes of partially hydrogenated vegetable oils are associated with elevated concentrations of inflammatory biomarkers like C-reactive protein, tumor necrosis factor-α, interleukin-6 and soluble intercellular adhesion molecule-1.[19] Further, it was reported that the CD4:CD8 T-lymphocytes ratio was increased by dietary *trans* fatty acids.[20]

The results of the present study enunciated the fact that cadmium was more potent in inducing damage and supplementation of vanaspati was of no ameliorative value in any of the toxic models. Further, the toxicities of CPS and cadmium were aggravated with the supplementation of vanaspati. Hence, it is concluded that consumption of vanaspati is harmful and leads to deleterious effects on health.
